# Evaluating brain functioning with NIRS in sports: Cerebral oxygenation and cortical activation are two sides of the same coin

**DOI:** 10.3389/fnrgo.2022.1022924

**Published:** 2022-11-18

**Authors:** Stéphane Perrey

**Affiliations:** EuroMov Digital Heath in Motion, Univ Montpellier, IMT Mines Ales, Montpellier, France

**Keywords:** hemodynamic responses, monitoring, cognitive function, health, physical performance

## Introduction

Neuroergonomics applies state-of-the-art methods and theories from neuroscience to understand how the human brain works in relation to cognitive and/or motor performance in everyday settings (Dehais et al., 2020). It is now well admitted that in ecological settings, near infrared spectroscopy (NIRS) makes possible the monitoring of cerebral oxygenation during the execution of motor tasks. In brief, NIRS is a non-invasive brain imaging method that measures concentration changes in oxygenated ([O_2_Hb]) and deoxygenated ([HHb]) hemoglobin of the brain, reflecting changes in cerebral perfusion and metabolism (Perrey, 2008). NIRS is a widely recognized method in measuring cerebral hemodynamic responses to multiple stimuli, such as changes in physiological parameters (e.g., blood pressure, oxygen, carbon dioxide) and neuronal activation (Scholkmann et al., 2022).

Brain NIRS studies can be divided into two main categories. Some studies have broadly used NIRS to assess cerebral oxygenation during critical care (Moerman and Wouters, 2010). Within this physiological context, hemoglobin saturation in the microvasculature is usually taken as an index of brain oxygenation. Thus, NIRS can be a clinical and research tool providing several medical and healthcare fields some insights into the hemodynamic response and cerebral oxygenation of the brain. In addition, brain functional activation studies using NIRS have opened the door to a new area of research in cognitive and behavioral neuroscience (Villringer et al., 1993). In this scenario, the NIRS method relies on the vascular response to a stimulus to capture the cortical neuronal activation. Functional NIRS (fNIRS) studies are based on the principle that changes in hemoglobin content and oxygenation are related to the regional cerebral blood flow (rCBF) following neural activity. In the neuroergonomics field, these two categories of studies collecting NIRS signals (i.e., hemodynamic evoked response and tissue oxygen saturation) assess the human brain functioning differently according to the tasks or conditions.

Thus, the NIRS method presents a unique opportunity to measure brain functioning in a continuous, real-time, and nonintrusive manner for multiple neuroergonomics scenarios, as those encountered in sports. Sports is a suitable but insufficiently underlined and undefined applied domain for neuroergonomics.

The first contributions to the field of brain NIRS in exercise and sports science were predominantly visible since the early 2000s, with an emphasis on the description of brain blood flow and oxygenation during exercise under hostile conditions (e.g., altitude in Ide and Secher, 2000; Subudhi et al., 2007) and on the role played by the brain in regulating cardiovascular responses and limitations to maximal exercise (Bhambhani et al., 2007; Dalsgaard and Secher, 2007; Rupp and Perrey, 2008). Three review papers synthesized the effects of whole-body exercise on brain hemodynamics measured by NIRS (Perrey, 2008; Ekkekakis, 2009; Rooks et al., 2011). During this period, only the oxygenation of the prefrontal cortex was the cortical area explored during incremental (Rooks et al., 2011) or supramaximal (Shibuya et al., 2004) cycling exhaustive exercises commonly conducted inside laboratory settings. Nowadays, current wireless and wearable fNIRS monitors that cover the entire brain (e.g., NIRSport, Brite) offer applicable tools in the sport sciences field but longitudinal and cohort studies are still needed to assess their use cases in sports. This opinion article concentrates on the benefits of utilizing brain NIRS for monitoring, i) the cerebral oxygenation status devoted to health issues when facing extreme conditions and trauma in sports, and ii) the induced cortical activation in real sporting situations, especially the mental demand. Behind the two main categories of studies using common NIRS methods, the underpinning principles for revealing either cerebral oxygenation or cortical activity are first recalled while indicating some derived markers. Second, feasibility and practical use of these NIRS-derived markers are presented through current proof-of-concept studies in sports. The technical and methodological details of NIRS are beyond the scope of this opinion but are well-presented elsewhere when movement and sports are concerned (Herold et al., 2017).

## NIRS demonstrates cerebral oxygenation

Cerebral oximetry based on NIRS is increasingly used in clinical settings such as during the perioperative period of cardiovascular operations or in neonatal intensive care (Obrig, 2014). As a continuous supply of oxygen is critical to the health of the brain, NIRS is often used to monitor the regional cerebral oxygen saturation (rScO_2_) of the frontal cortex. Herein, the objective is to identify degraded adjustments in cerebral oxygenation, and then identify countermeasures to reverse cerebral oxygenation responses and thereby prevent any long-term sequels. Global brain oxygenation through rScO_2_ appears a smart approach that the neuroergonomics field including sports can further benefit from. RScO_2_, or sometimes called the tissue oxygenation/saturation index depending on the NIRS manufacturer, is defined as the ratio (%) of O_2_Hb to total hemoglobin (which is the sum of [O_2_Hb] and [HHb]). The NIR light is absorbed by HHb and O_2_Hb in both arterial and venous vessels, but rScO_2_ reflects mainly cerebral venous oxygen saturation (Watzman et al., 2000). Concerning its validation, NIRS-monitored cerebral oxygenation for different oximeters was found in good agreement with oxygen saturation in the jugular vein (Webber, 1996; la Cour et al., 2018). Finally, rScO_2_ is derived from a spatially-resolved spectroscopy-NIRS system (Wolf et al., 2007). Here, the multi-distance configuration allows the absolute measurement of cerebral oxygenation that can be compared across individuals and over time and is more robust against motion artifacts and less influenced by systemic physiological interferences. One of the main situations of interest for monitoring rScO_2_ lies on specific physiological conditions that can impair the cerebral perfusion and thus the cerebral autoregulation; being defined as the ability to maintain steady state cerebral perfusion and oxygenation when blood pressure fluctuates (Paulson et al., 1990). Continuous NIRS monitoring of cerebral oxygenation with potential reference values can be considered in several real sporting situations as outlined thereafter. Noteworthy, the (patho)physiological conditions affecting cerebral oxygenation require simultaneous recording of multiple physiological signals evolving during exercise (e.g., blood pressure, heart rate, partial pressure of CO_2_) in providing an accurate interpretation of the NIRS signals (Scholkmann et al., 2022).

## fNIRS demonstrates cortical activation

NIRS also measures cerebral hemodynamic changes due to cortical neuronal activation. The term “activation” has been operationally defined by the focal increase in rCBF for all neuroimaging methods based on the vascular response to a stimulus; a decrease in rCBF being termed “deactivation” (Obrig and Villringer, 2003). The hemodynamic changes with NIRS to assess the functional cortical activation is based on the assumption that a given stimulus (e.g., motor, cognitive) will induce a neuronal response, which in turn, triggers local arterial vasodilation, with an increase in cerebral blood volume and rCBF, a process known as neurovascular coupling (Perrey, 2008). Thus, a cerebral region is considered active when its rCBF increases, producing a canonical hemodynamic response (i.e., increase in [O_2_Hb] and decrease in [HHb], Obrig et al., 1996). This last decade, there has been an increase in functional activation studies with fNIRS in the sport sciences field (Herold et al., 2017; Perrey and Besson, 2017). One important issue to consider for such functional activation studies is what experimental setup to adopt in order to exhibit the expected hemodynamic response. In general, there are two types of design: block design and event-related design. Block designs are the usual paradigms implemented in fNIRS studies. The duration of the rest period should allow the stimulus-evoked hemodynamic response to build up and return to the baseline level (Franceschini et al., 2003). Block design investigates the pattern of brain activation across one or more blocks alternating resting and task conditions. It often includes more than five trials lasting 20–30 s per condition. Alternately, event-related design investigates brain activation triggered by events occurring in a random order (Schroeter et al., 2002). Event-related design is able to measure changes in brain activity over short and discrete intervals. Hence, this more ecological design allows for detecting transient changes in hemodynamic responses. However, event-related design usually needs an important number of trials in order to enhance the statistical power in detecting a reliable hemodynamic response. Tracking cortical oxygenation is reported in real sporting situations below.

## Current use of cerebral NIRS during sports

The ability of NIRS devices to perform real measurements of brain oxygenation can be proper for monitoring the fitness level in individuals at regular time intervals. Recently, Herold et al. (2020) emphasized that brain activity derived from fNIRS measures (e.g., rScO_2_) could be used as a valuable and promising marker of internal burden (fatigue, stress) during physical exercise. Authors highlighted first thoughts on the practical application of brain-derived parameters to prescribe exercise intensity in endurance sports. To date, it is unclear to what extent NIRS changes are influenced by sport-related factors such as risk of contact exposure and concussion history (Churchill et al., 2017), environment of practice (altitude, acceleration force) and training exertion features.

First, disturbances in cerebral perfusion and oxygenation are major markers for mild traumatic brain injury as encountered in sport-related concussion. After concussion, individuals with persisting symptoms show acute decreased brain oxygenation patterns in frontal cortices when compared to healthy individuals during postural tasks (Helmich et al., 2016). This brain-behavior diagnosis can be appropriate for assessing the return-to-play following concussion. In addition, NIRS is able to monitor the long-term neuropsychological impairments affecting anxiety, problem solving, planning, memory, attention, concentration, and behavior following recurrent concussions in multiple contact sport categories such as boxing, snowboarding, rugby, and football. Measuring the relative changes in O_2_Hb and HHb in the prefrontal cortex in response to hypercapnia (block design with 5 repeated 20 s breath holds) was found to be a viable biomarker in a cost-effective manner to assess the concussion recovery timeline for up to 14 days (Bishop and Neary, 2018). In the same vein, changes in brain health due to long-term adaptations to a regular exercise regimen are associated with enhanced rCBF and brain activation (Perrey, 2013) but sustained exercise exertion over time may negatively impact brain functioning (Blain et al., 2019). This opens up the feasibility of proposing a follow-up of brain changes (i.e., O_2_Hb and HHb) using cerebral fNIRS in the study of optimal training load and long-term effects on brain health, along with the early detection of overtraining.

Second, impaired cerebral oxygenation (i.e., imbalance between cerebral oxygen supply and demand) appears during hypoxic and/or acceleration force exposures in sports. Evaluating changes in brain oxygenation under extreme conditions in humans is challenging but needed. Studies reported large changes in brain oxygenation states caused by exposure to multiple and high acceleration forces in pilots during parabolic flight (Schneider et al., 2013), flight missions (Kobayashi and Miyamoto, 2000), and aerobatic flight (Fresnel et al., 2021). The same likely applies to Formula One drivers experiencing 5–6 g while braking and 2–7 g while cornering. The high sensitivity of NIRS signals to acceleration force point out strong variations in the status of cerebral oxygenation, revealing occurrences of short hypoxia or hyperoxia epochs leading potentially to G-induced loss of consciousness and other deleterious symptoms. Further, cognitive processes associated with the best visuo-spatial abilities (i.e., neural efficiency) could be unveiled by fNIRS in the brain of highly skilled aerobatic pilots and Formula One race-car professional drivers. Finally, assessing cortical activation and tracking cerebral oxygenation changes during ascent and stay at high altitude environments may inform both on the neural correlates of cognitive control (Davranche et al., 2016; Champigneulle et al., 2022) and in the pathogenesis of acute mountain sickness (Manferdelli et al., 2021), respectively.

Neuroergonomics is significantly associated with mental demand and fatigue. Studying cognitive demand using brain imaging techniques in uncontrolled environments is typically encountered in sports (Perrey, 2022). Recent pilot studies have carried out, for the first-time, online measurements of hemodynamic response changes with fNIRS during sports-relevant motor behavior (e.g., basketball dribbling skills in Carius et al., 2020) by using a dedicated block design. Here, assessing brain activity during complex movements aims to be a prerequisite for considering brain-behavior relationships and enhancing motor performance. Regarding psychological factors, the fNIRS study of Slutter et al. (2021) showed that the prefrontal cortex and the left temporal cortex were pivotal brain areas in soccer players who were more anxious and missed penalties, supporting evidence for the neural efficiency theory.

## Challenges and potentials

To obtain meaningful data in sports, it is important to cope with the influences of artifacts and to develop appropriate signal processing. To date, further efforts need to be performed to face the current methodological limitations when collecting NIRS data with wearable and wireless brain devices (e.g., Brite23) during functional tasks, as suggested in the literature (Herold et al., 2017; Yücel et al., 2021). Regarding fNIRS applications in sports, two main concerns are proposed in order to improve the quality and provide the correct interpretation of the NIRS signals collected during physical exercise. Essentially, movement artifacts should be approached by carefully controlling the experimental environment whenever possible. Importantly, assessing the optode-scalp contact “quality” throughout the exercise duration is thought to be a first priority. Within this context, collecting additional data, such as camera images (Bang et al., 2013) or with an accelerometer (Virtanen et al., 2011) should be considered. It means that the primary objective of an ideal fNIRS cap is to continuously stabilize the optodes over the scalp by identifying an optimal comfort pressure threshold. An inflatable pneumatic/vacuum cap design (Kassab et al., 2015) despite some technological challenges provides interesting and promising solutions to apply into the field of sports. With long-term monitoring, issues pertaining to the effect of sweat and heat on the collected signals are also important ones. Further, video can help to identify key events to time-lock the acquired fNIRS signals in ecological conditions. Recently, Pinti et al. (2017) developed automatic identification of a functional events algorithm to assess the functional brain activation. When exercising, systemic physiological fluctuations (e.g., in heart rate, arterial CO_2_ concentration, blood pressure, skin perfusion, and more) can interfere significantly with brain NIRS measurements. In addition to removing the influence of systemic physiological changes originating at the more superficial layers of the head by using short-separation channels, a multimodal physiology data monitoring approach synchronized to fNIRS signals (e.g., Strangman et al., 2018) appears as a second priority. All these measurements can be used in signal processing methods to isolate and properly remove the non-neuronal-related functional responses (Yücel et al., 2021; Scholkmann et al., 2022).

## Conclusion

NIRS represents an effective brain monitoring modality and has become an emergent ideal tool for studying brain functioning in sport environments. Recent studies in applied physiology and neuroscience show well the applicability of NIRS to investigate brain functioning in various and complex sport-related situations. NIRS provides some viable biomarkers related to cerebral oxygenation and cortical activation for assessing the neurophysiological state of the brain when exercising. The current availability of wearable NIRS systems and their robustness to motion artifacts make them a serious candidate for brain functioning measures on individuals performing cognitive-motor tasks. Hence, the sports domain should guide our future work in the neuroergonomics field.

## Author contributions

The author confirms being the sole contributor of this work and has approved it for publication.

## Conflict of interest

The author declares that the research was conducted in the absence of any commercial or financial relationships that could be construed as a potential conflict of interest.

## Publisher's note

All claims expressed in this article are solely those of the authors and do not necessarily represent those of their affiliated organizations, or those of the publisher, the editors and the reviewers. Any product that may be evaluated in this article, or claim that may be made by its manufacturer, is not guaranteed or endorsed by the publisher.
